# Regulation of TIR-1/SARM-1 by miR-71 Protects Dopaminergic Neurons in a *C. elegans* Model of LRRK2-Induced Parkinson’s Disease

**DOI:** 10.3390/ijms25168795

**Published:** 2024-08-13

**Authors:** Devin Naidoo, Alexandre de Lencastre

**Affiliations:** 1 Frank H. Netter MD School of Medicine, Quinnipiac University, North Haven, CT 06473, USA; danaidoo@quinnipiac.edu; 2 Department of Biological Sciences, Quinnipiac University, Hamden, CT 06518, USA

**Keywords:** miRNA, elegans, Parkinson, neurodegeneration, aging

## Abstract

Parkinson’s disease (PD) is a common neurodegenerative disorder characterized by symptoms such as bradykinesia, resting tremor, and rigidity, primarily driven by the degradation of dopaminergic (DA) neurons in the substantia nigra. A significant contributor to familial autosomal dominant PD cases is mutations in the LRRK2 gene, making it a primary therapeutic target. This study explores the role of microRNAs (miRNAs) in regulating the proteomic stress responses associated with neurodegeneration in PD using *C. elegans* models. Our focus is on miR-71, a miRNA known to affect stress resistance and act as a pro-longevity factor in *C. elegans*. We investigated miR-71’s function in *C. elegans* models of PD, where mutant LRRK2 expression correlates with dopaminergic neuronal death. Our findings reveal that miR-71 overexpression rescues motility defects and slows dopaminergic neurodegeneration in these models, suggesting its critical role in mitigating the proteotoxic effects of mutant LRRK2. Conversely, miR-71 knockout exacerbates neuronal death caused by mutant LRRK2. Additionally, our data indicate that miR-71’s neuroprotective effect involves downregulating the toll receptor domain protein *tir*-1, implicating miR-71 repression of *tir*-1 as vital in the response to LRRK2-induced proteotoxicity. These insights into miR-71’s role in *C. elegans* models of PD not only enhance our understanding of molecular mechanisms in neurodegeneration but also pave the way for potential research into human neurodegenerative diseases, leveraging the conservation of miRNAs and their targets across species.

## 1. Introduction

Parkinson’s disease (PD) is an aging-associated neurodegenerative movement disorder characterized by the loss of dopaminergic neurons in the substantia nigra [1]. The corresponding loss of nigrostriatal pathway dopamine signaling, which is required for movement stimulation, leads to resting tremor, muscular rigidity, and bradykinesia [1]. Although the exact cause of PD is unknown, many risk factors have been identified, most notably, aging, as well as several genes that can cause rare familial forms of PD [2].

Mutations in the leucine-rich repeat kinase 2 (*LRRK2*) gene are the most frequent cause of late-onset, autosomal dominant PD and are largely indistinguishable from sporadic disease [3]. The *LRRK2* gene encodes for a large protein (2,527-amino acids) with three enzymatic regions: the Ras of complex (ROC) GTPase domain, the carboxy-terminal of ROC (COR) domain, and the kinase domain [4]. Though the specific function of the LRRK2 protein is still largely unknown, it has been shown to have a wide number of roles in the nervous system [5,6]. At least 16 known missense mutations in the enzymatic regions of *LRRK2* have been shown to be associated with PD, with the most prevalent of these mutations being the G2019S mutation in the kinase domain [7,8]. Additionally, the expression of G2019S *LRRK2* in model organisms such as *C. elegans* have been shown to cause age-dependent dopaminergic neurodegeneration [9,10,11]. Though the G2019S mutation has been shown to significantly increase kinase activity leading to neuronal damage, the exact pathological mechanism has yet to be discovered [12].

Recently, it has been shown that lifespan extension through multiple pathways, such as insulin/insulin-like growth factor-1 (IGF-1) signaling, target of rapamycin (TOR), and mitochondrial respiration, can be neuroprotective in the G2019S LRRK2 model of *C. elegans* [11]. In a survey of *C. elegans* miRNAs that are involved in aging and development, miR-71 has been shown to increase lifespan and stress response activation (heat shock and oxidative stress) [13]. Evaluation of the miR-71 spatiotemporal expression during aging showed that miR-71 increased expression most dramatically in head neurons [13]. Additionally, miR-71 expression in neurons is sufficient in extending the lifespan of *C. elegans*, at least partially, by regulating the transcription factor DAF-16, which is involved in the insulin signaling pathway associated with longevity [14]. These observations led us to evaluate the possible protecting roles of miR-71 in the stress response in a model of neurodegenerative disease. Here, we evaluate the neuroprotective effects of lifespan-extending miRNA, miR-71, in the G2019S LRRK2 worm model.

In neuronal models of *C. elegans*, miR-71 has been shown to target the gene for Toll/interleukin-1 receptor domain protein (*tir*-1) in olfactory neurons to promote proteostasis and prevent neurodegeneration [15,16]. TIR-1 is a homolog of mammalian sterile alpha and TIR motif-containing 1 (SARM1), with functions in innate immunity and the clearance of damaged neurons, and it contains a NADase domain that cleaves nicotinamide adenine dinucleotide (NAD^+^) [17]. In response to both pathogen- and non-pathogen-associated cellular stress, *tir*-1 can oligomerize to form a multimeric protein complex that activates its NAD^+^ glycohydrolase activity [18]. The depletion of NAD^+^ in response to the activation of *tir*-1 leads to enhanced axonal degeneration in both mammalian and *C. elegans* models [19]. Additionally, *tir*-1 has the ability to activate the mitogen-activated protein kinase (MAPK) signaling cascade in response to injury, which can inhibit axon regeneration and promote degeneration [20]. In humans, SARM1-mediated axon degeneration has been implicated in multiple neurodegenerative disorders, including PD [21]. Thus, the current development of treatments for neurodegenerative disorders has focused on targeting and inhibiting SARM1 [21]. Here, we evaluate the neuroprotective effects of miR-71 in *C. elegans* models of LRRK2-induced PD and whether miR-71 targets *tir*-1 in conferring protection against dopaminergic neurodegeneration. Our data demonstrates that miR-71 regulates *tir*-1 to attenuate LRRK2-induced neurodegeneration.

## 2. Results

### 2.1. miR-71 Rescues Loss of Dopaminergic Neurons

In order to assess the role of miR-71 on the dysfunctional pathology associated with mutant LRRK2 expression, a dopaminergic neurodegeneration assay was conducted to investigate the effects of miR-71 in the LRRK2 *C. elegans* PD models. Yao and colleagues showed that expression of human LRRK2 PD-linked mutants in *C. elegans* dopaminergic neurons induce neurodegeneration early in adulthood [10]. LRRK2 strains express G2019S PD-linked mutant human LRRK2 under the control of the pDat-1 dopaminergic neuron promoter with a GFP fluorescent marker (pDat-1::LRRK2(G2019S); pDat-1::GFP). A GFP control strain expresses only the GFP fluorescent marker under the same pDat-1 promoter (pDat-1::GFP line). Double mutants between G2019S and miR-71 overexpression ([Pdat-1::LRRK2(G2019S); pDat-1::GFP]; miR-71 O/E (nls286); sur-5::GFP) and G2019S and miR-71 knockout ([Pdat-1::LRRK2(G2019S); pDat-1::GFP]; miR-71 KO (n4115)) were created to assess the effects of miR-71 in the LRRK2(G2019S) PD model. Additionally, double-mutants between GFP control strains and miR-71 overexpression (pDat-1::GFP; miR-71 O/E (nls286); sur-5::GFP) and GFP control strains and miR-71 knockout (pDat-1::GFP; miR-71 KO (n4115)) were created to assess the effects of miR-71 on healthy dopaminergic neurons.

**Figure 1:**
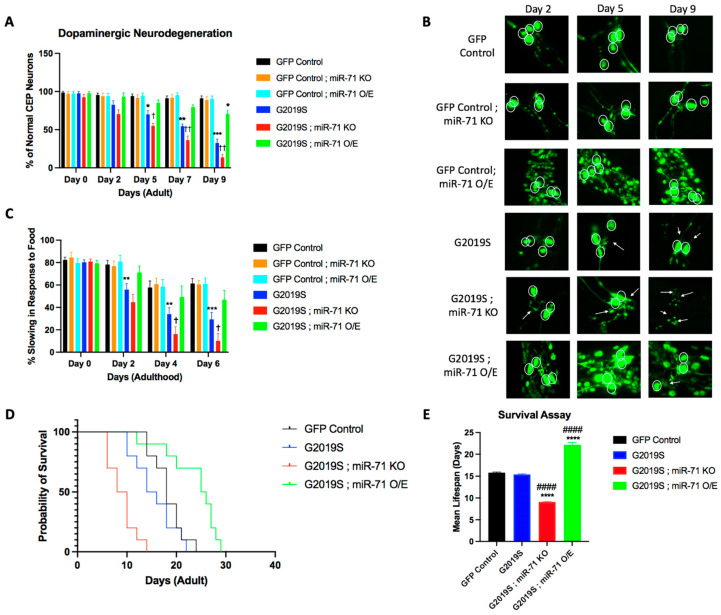
miR-71 preserves dopaminergic neurons in LRRK2 worms. (**A**) Results from the dopaminergic neurodegeneration assay at different time points of adulthood (*n* = 10 worms). Error bars indicate SEM. One-way ANOVA: * *p* < 0.05, ** *p* < 0.01, *** *p* < 0.001 versus GFP control worms at each time point. † *p* < 0.05 and †† *p* < 0.01 versus G2019S worms at each time point. (**B**) Z-stack images from data shown in (**B**) were merged to identify DA neuronal cell bodies. Circles denote DA neurons and white arrows indicate abnormal or absent cephalic dopaminergic neurons. (**C**) Results from the basal slowing assay at different time points of adulthood (*n* = 20 worms). Error bars indicate SEM. One-way ANOVA: ** *p* < 0.01 and *** *p* < 0.005 versus GFP control worms at each time point. † *p* < 0.05 versus G2019S worms at each time point. (**D**) Kaplan–Meier survival analysis comparing LRRK2 expressing worms crossed with miR-71 mutants to GFP Control models. (E) Mean lifespan from data shown in (**D**). Bars represent mean ± SEM. One-way ANOVA: **** *p* < 0.0005 versus GFP Control. ^####^ *p* < 0.0005 versus G2019S.

Our results of G2019S and miR-71 double mutants show that miR-71 overexpression rescues the loss of dopaminergic neurons exhibited by *C. elegans* expressing PD-linked LRRK2 mutants (Figure 1A,B). Conversely, the knockout of miR-71 accelerated the neurodegeneration of dopaminergic neurons expressing mutant G2019S LRRK2. Neither knockout nor overexpression of miR-71 alone had any effect on the viability of healthy dopaminergic neurons in the control strain that expresses GFP but not LRRK2. These results suggest that miR-71 is required for and rescues dopaminergic neuron viability in response to G2019S LRRK2-induced neurotoxicity.

Prior studies have also shown that the ability of worms to sense bacterial food is a dopamine-specific behavior that causes the worms to slow down their movement in the presence of food, which is termed “basal slowing” [22]. Yao and colleagues showed that the expression of human LRRK2 PD-linked mutants in *C. elegans* dopaminergic neurons caused a loss in basal slowing behavior [10]. Given that the basal slowing response in *C. elegans* is controlled by the dopaminergic system, we aimed to test if the miR-71 rescue effects on dopaminergic (DA) neurons would also rescue basal slowing behavior.

Our results of G2019S and miR-71 double mutants show that miR-71 overexpression rescues the loss of basal slowing exhibited by *C. elegans* expressing PD-linked LRRK2 mutants in dopaminergic neurons (Figure 1C). Beginning on day 2 and persisting through the time course of the experiment, the LRRK2 mutant strain (G2019S) exhibits a significant decrease in basal slowing as compared to the GFP control strain (Figure 1C). Double mutants that overexpress miR-71 (G2019S; miR-71 OE) retain normal basal slowing behavior, comparable to GFP control worms, at every time point. Conversely, we found that knockout of miR-71 (G2019S; miR-71 KO) induces an acceleration of the loss of basal slowing behavior seen in G2019S mutant strains. Differences in baseline motility of LRRK2 worms in the absence of food was found to be insignificant (Supplementary Figure S1). These data suggest that miR-71 is required to attenuate the pathogenic effects caused by mutant LRRK2 expression and that overexpression of miR-71 can fully rescue the normal basal slowing response, making it indistinguishable from wild-type controls. Additionally, neither miR-71 knockout nor overexpression had effects on basal slowing behavior in GFP Control worms, showing that miR-71’s effects are specific to the organismal response to G2019S LRRK2-induced neurotoxicity.

Previous studies by de Lencastre and colleagues have shown that miR-71 is important for the longevity of *C. elegans* [13]. Since miR-71 mutations alter the *C. elegans* lifespan, we conducted a survival assay to test the lifespan of G2019S strains and compare them to miR-71 double-mutant worms (Figure 1D). As expected, G2019S;miR-71 KO double-mutant worms experienced a significant decrease in lifespan, while the G2019S;miR-71 overexpression strain experienced an increase in longevity (Figure 1E), consistent with previous results [13,14,23]. Importantly, the G2019S single-mutant worms did not show a significant difference in longevity compared to GFP control worms. This suggests that the expression of the mutant LRRK2 protein in *C. elegans* alone does not alter the longevity of the worms and that, therefore, the neuroprotective effects of miR-71 could be linked to its effects on longevity.

### 2.2. Loss of tir-1 Reverses Effects of miR-71 Knockout

**Figure 2:**
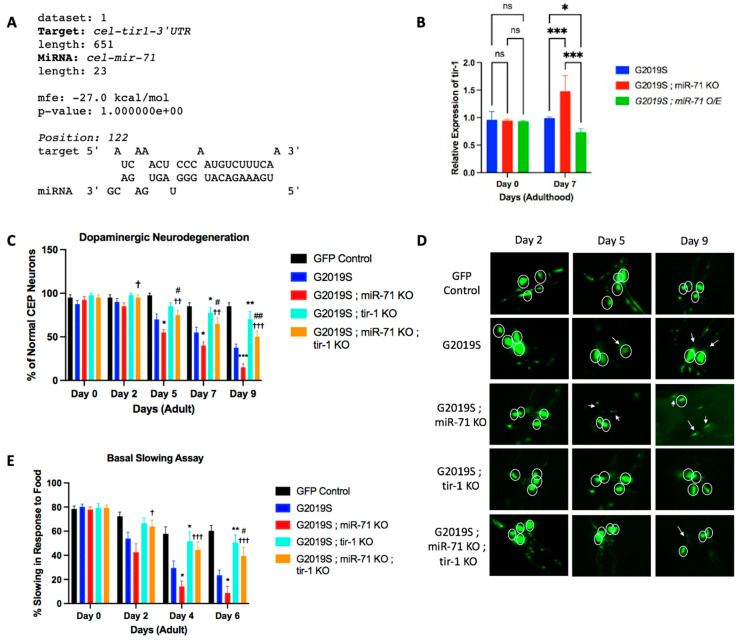
Loss of the toll-like receptor *tir*-1 is neuroprotective in LRRK2 expressing *C. elegans.* (**A**) The putative binding site between miR-71 and the toll-like receptor *tir*-1 3′ untranslated region (UTR). The minimum free energy of binding is −27.0 kcal/mol. (**B**) Results from qRT-PCR looking at relative expression of *tir*-1 at different time points of adulthood (*n* = 3 biological replicates). Error bars indicate SEM. One-way ANOVA: ns (not statistically significant), * *p* < 0.05, and *** *p* < 0.005. (**C**) Results from the dopaminergic neurodegeneration assay at different time points of adulthood (*n* = 10 worms). Error bars indicate SEM. One-way ANOVA: * *p* < 0.05, ** *p* < 0.01, *** *p* < 0.001 versus G2019S worms at each time point. † *p* < 0.05, †† *p* < 0.01, ††† *p* < 0.005 versus G2019S; miR-71 KO at each time point. # *p* < 0.05 and ## *p* < 0.01 versus GFP control at each time point. (**D**) Z-stack images from data shown in C were merged to identify DA neuronal cell bodies. Circles denote DA neurons and white arrows indicate abnormal or absent cephalic dopaminergic neurons. (**E**) Results from the basal slowing assay at different time points of adulthood (*n* = 20 worms). Error bars indicate SEM. One-way ANOVA: * *p* < 0.05 and ** *p* < 0.01 versus G2019S at each time point. † *p* < 0.05 and ††† *p* < 0.005 versus G2019S; miR-71 KO at each time point. # *p* < 0.05 versus GFP control at each time point.

To investigate the molecular mechanisms for the miR-71 rescue of LRRK2-induced neurodegeneration, we tested the potential gene targets of miR-71. We conducted a survey of candidate miR-71 targets across organisms and considered *tir*-1 as a high-probability candidate in *C. elegans* neurons [24]. The toll-like receptor, TIR-1, has been shown to be important for modulating neuronal and axonal degradation in *C. elegans*, likely through direct binding between miR-71 and the *tir*-1 3′ untranslated region (UTR) [15,16]. Examination of the *tir*-1 3′UTR shows that it contains a high-probability consensus binding site for miR-71 consisting of an 8-mer A1 (adenosine complementary to nucleotide 1 of a miRNA) in the seed sequence and a significant binding with the anchor sequence (Figure 2A). The predicted 8-mer A1 binding structure represents the strongest binding capability of miRNAs to their target 3′ UTR with the adenosine positioned complementarily to nucleotide 1 of the miRNA believed to aid in Argonaute protein recognition [25]. In addition, the low free energy of binding, −27.0 kcal/mol, presents an energetically favorable binding between miR-71 and the 3′ UTR of *tir*-1 (Figure 2A).

Knowing that miR-71 has the capability to bind the *tir*-1 3′ UTR, qRT-PCR was conducted to analyze the level of expression of *tir*-1 in miR-71 mutant worms. By day 7 of adulthood, G2019S;miR-71 KO double mutants had an increased expression of *tir-1* compared to G2019S single mutant worms (Figure 2B). Conversely, G2019S;miR-71 OE had a decreased expression of *tir*-1 (Figure 2B). The decline in *tir*-1 expression in G2019S;miR-71 OE animals coincides with a 2.5-fold increase of miR-71 at day 7 of adulthood in this strain (Supplementary Figure S2). These results are consistent with a model wherein miR-71 negatively regulates *tir*-1 mRNA levels.

Since miR-71 levels are inversely correlated with *tir*-1 expression, suggesting that miR-71 negatively regulates *tir*-1, we then tested if miR-71 functionally regulates *tir*-1 function in LRRK2-induced pathology. Loss-of-function *tir*-1 mutants have been previously shown to suppress neuron degeneration in models of neurodegenerative disease and neuronal injury [20,26]. We have shown here that miR-71 KO causes the acceleration of LRRK2-induced pathology. We therefore reasoned that if miR-71 negatively regulates *tir*-1, a *tir*-1 loss-of-function mutation would suppress the miR-71 KO phenotype in an LRRK2 mutant strain. We generated a G2019S; *tir*-1 knockout double-mutant strain ([Pdat-1::LRRK2(G2019S); pDat-1::GFP]; *tir*-1 KO (qd4)) and G2019S;miR-71 KO;*tir*-1 knockout triple-mutant strain ([Pdat-1::LRRK2(G2019S); pDat-1::GFP]; miR-71 KO (n4115); *tir*-1 KO (qd4)) and compared their neurodegeneration and basal slowing response to G2019S and G2019S; mir-71 KO strains (Figure 2C–E). Our results show that the knockout of *tir*-1 suppresses the dopaminergic neuron death exhibited in G2019S worms, as expected (Figure 2C).

Additionally, we show that the knockout of *tir*-1 in the triple-mutant models suppresses the accelerated dopaminergic neuron death exhibited in double-mutant G2019S;miR-71 KO worms (Figure 2C). Beginning on day 2 of adulthood, the *tir*-1 knockout triple-mutant strain (G2019S;miR-71 KO;*tir*-1 KO) began exhibiting a significant decrease in DA neurodegeneration as compared to the G2019S;miR-71 knockout double-mutant strain (Figure 2C). By day 9, the *tir*-1 knockout triple-mutant strain (G2019S;miR-71 KO;*tir*-1 KO) showed a significant preservation of dopaminergic neurodegeneration as compared to the double-mutant G2019S;miR-71 knockout worms, showing that *tir*-1 knockout suppresses the LRRK2-mediated neurodegeneration in miR-71 KO mutants. It should be noted that this rescue was not complete as there is still a significant increase in the DA neurodegeneration of *tir*-1 triple-mutants (G2019S;miR-71 KO;*tir*-1 KO) as compared to GFP control worms, suggesting that, perhaps, other genes beyond *tir*-1 might also functionally interact with miR-71 (Figure 2C). Overall, the finding that knockout of *tir*-1 can partially protect dopaminergic neurons in LRRK2-induced neurodegeneration in miR-71 knockout models shows evidence of genetic epistasis between miR-71 and *tir*-1 and is consistent with a model wherein miR-71 protects from LRRK2-mediated neurodegeneration by functioning through *tir*-1.

Having shown that the knockout of *tir*-1 can partially rescue the neurodegeneration of dopaminergic neurons in *C. elegans* expressing mutant LRRK2, we sought to examine if the same effects would be seen in basal slowing behavior. We found that the G2019S;mir-71 KO;*tir*-1 KO triple-mutant strain indeed rescues basal slowing behavior back to or similar to wild-type level (Figure 2E). Although on day 6, this rescue in the basal slowing behavior of *tir*-1 knockout triple mutants (G2019S;miR-71 KO;*tir*-1 KO) as compared to GFP control worms is not complete, the triple mutants show significantly enhanced basal slow as compared to G2019S or G2019S;miR-71 KO worms alone (Figure 2E). Just like the rescue observed in neurodegeneration, these data similarly show that miR-71 attenuates the pathogenic effects caused by LRRK2 expression, at least partially, through its downstream target, *tir*-1.

### 2.3. miR-71 Regulates tir-1 to Rescue Dopaminergic Neurons

**Figure 3:**
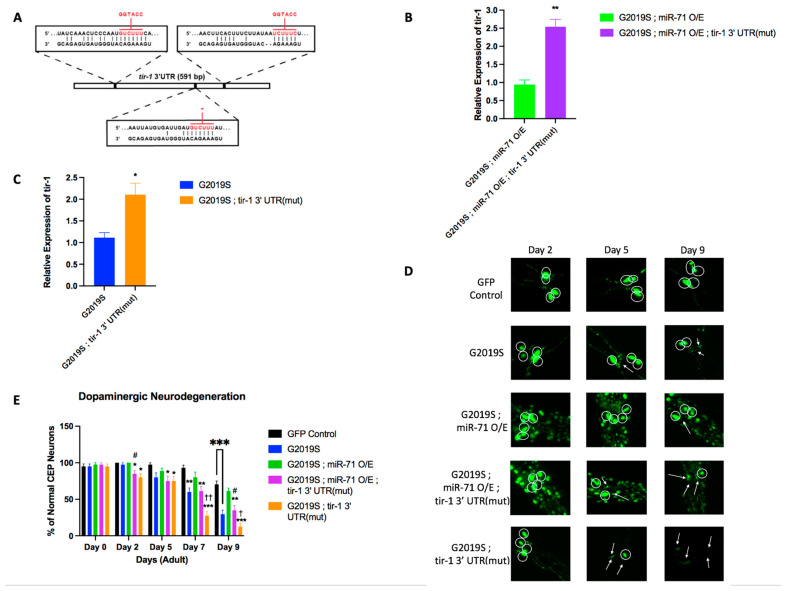
miR-71 directly regulates the toll-like receptor *tir*-1 in LRRK2 expressing *C. elegans.* (**A**) Three miR-71 binding sites on the 3′ UTR of *tir-1* were mutated to remove miR-71 regulation capability (tir-1 3′UTR(mut)). (**B**,**C**) Results from qRT-PCR looking at relative expression of *tir*-1 at different time points of adulthood (*n* = 3 biological replicates). Experiments for qRT-PCR were done in triplicate with about 50 worms per strain per experiment. Error bars indicate SEM. One-way ANOVA: * *p* < 0.05 and ** *p* < 0.01. (**D**) Z-stack images from data shown in B were merged into a singular image to identify DA neuronal cell bodies. Circles denote DA neurons and white arrows indicate abnormal or absent cephalic dopaminergic neurons. (**E**) Results from the basal slowing assay at different time points of adulthood (*n* = 20 worms). Error bars indicate SEM. One-way ANOVA: * *p* < 0.05, ** *p* < 0.01, *** *p* < 0.001 versus GFP control worms at each time point. † *p* < 0.05 and †† *p* < 0.01 versus G2019S at each time point. # *p* < 0.05 versus G2019S;m71 OE at each time point.

In order to confirm that miR-71 is directly regulating *tir*-1 mRNA in the LRRK2(G2019S) expressing models, we utilized a strain that contains a mutation in all three predicted *miR-71* binding sites in the 3′ UTR of *tir*-1 (*tir*-1 3′UTR(mut)) (Figure 3A) [15]. These *tir*-1 3′ UTR(mut) worms were crossed with the previously generated G2019S;miR-71 OE mutants ([Pdat-1::LRRK2(G2019S); pDat-1::GFP]; miR-71 O/E (nls286); sur-5::GFP; *tir*-1 3′UTR(mut)) and G2019S worms ([Pdat-1::LRRK2(G2019S); pDat-1::GFP]; *tir*-1 3′UTR(mut)).

To test the effects of the *tir*-1 3′UTR mutation on *tir*-1 mRNA expression, qRT-PCR was conducted to analyze the level of expression of *tir*-1 in day 7 adult mutant worms. The mutation of miR-71 binding sites on the *tir*-1 3′UTR led to a nearly 2-fold increase of *tir*-1 expression in both G2019S and G2019S;miR-71 OE strains (Figure 3B,C). The increase in *tir*-1 expression in G2019S;miR-71 OE;*tir*-1 3′UTR(mut) animals coincides with a 2.5-fold increase of miR-71 at day 7 of adulthood in this strain (Supplementary Figure S3). These results are consistent with a model wherein miR-71 negatively regulates *tir*-1 mRNA levels in the context of LRRK2(G2019S) neurodegeneration.

Knowing that the loss of *tir*-1 partially rescued dopaminergic neuron loss, we examined if the deletion of miR-71 binding sites on the *tir*-1 3′UTR might accelerate DA neurodegeneration. We saw that beginning on day 2 of adulthood, the G2019S;*tir*-1 3′UTR(mut) and G2019S;m71 OE;*tir*-1 3′UTR(mut) began exhibiting a significant increase in DA neurodegeneration (Figure 3 D,E). By day 9, the mutation of *tir*-1 3′UTR (G2019S;*tir*-1 3′UTR(mut) and G2019S;m71 OE;*tir*-1 3′UTR(mut) showed a significant acceleration in dopaminergic neurodegeneration compared to G2019S and double-mutant G2019S;miR-71 OE worms, respectively. We also noted that G2019S;miR-71 O/E;*tir*-1 3′UTR(mut) animals showed a higher number of healthy CEP neurons as compared to G2019S;*tir*-1 3′UTR (mut) animals, potentially suggesting that miR-71 may have additional targets that promote neuroprotection. Together, these results show that the deletion of the *tir*-1 3′UTR miR-71 binding sites did, indeed, lead to an acceleration in dopaminergic neuron death in G2019S strains, suggesting that miR-71 directly regulates *tir*-1 (Figure 3E). In addition, G2019S;miR-71OE;*tir*-1 3′UTR(mut) did not show a rescue of dopaminergic neuron loss, indicating that the rescue effect of miR-71 OE depends on the presence of miR-71 binding sites on the *tir*-1 3′UTR. Overall, these data confirm that the miR-71 regulation of *tir*-1 is partially responsible for the protection of dopaminergic neurons from mutant LRRK2-induced neurodegeneration.

## 3. Discussion

Our study provides significant insights into the neuroprotective role of miR-71 in *C. elegans* in the context of LRRK2-induced Parkinson’s disease. The LRRK2 gene, particularly its G2019S mutation, is a well-established contributor to PD pathogenesis in humans [27]. Our findings suggest that miR-71 plays a crucial role in mitigating the deleterious effects of LRRK2 mutations in *C. elegans* models, particularly in dopaminergic neurons, which are central to PD pathology.

The overexpression of miR-71 in our *C. elegans* model of LRRK2-induced PD significantly preserved dopaminergic neuron viability and attenuated neurodegeneration. Notably, miR-71 overexpression not only delayed the onset of neurodegeneration but also enhanced the lifespan of the affected animals. Prior studies have uncovered the role of miR-71 in directly repressing *tir*-1 expression through binding to the complementary site on the *tir*-1 3′ UTR [15,16]. This interaction between miR-71 and *tir*-1 has been shown to be important for maintaining proteostasis and preventing neurodegeneration in olfactory neurons [15,16]. However, our results indicate a novel interaction between miR-71 and the toll-like receptor gene, *tir*-1, in a *C. elegans* disease model expressing LRRK2 in dopaminergic neurons. The attenuation of neurodegenerative effects through miR-71 overexpression correlates with a decrease in *tir*-1 expression. Additionally, the loss of neuroprotection by mutating miR-71 binding sites on the *tir*-1 3′ UTR suggests that miR-71 may exert its neuroprotective effects, at least partly, by downregulating *tir*-1. However, the incomplete rescue in *tir*-1 knockout mutants indicates that miR-71′s protective mechanism is not solely dependent on *tir*-1 modulation.

Since *tir*-1 is implicated in innate immunity and inflammation, its downregulation by miR-71 might signify a strategy to reduce neuroinflammation, a key component of PD pathogenesis [27,28]. In *C. elegans*, *tir*-1 can undergo a phase transition in response to stress that enhances the NAD^+^ glycohydrolase activity of *tir*-1, which is crucial for activating the p38 PMK-1 immune pathway to induce degeneration [17,29]. In models of amyotrophic lateral sclerosis (ALS) in *C. elegans*, the activation of *tir*-1 in response to mutated proteins associated with ALS in neurons is necessary for neurodegeneration [26]. In parallel, the mutant LRRK2 proteins associated with PD may function in a similar manner to activate the NAD^+^ glycohydrolase function of *tir*-1 in *C. elegans* to induce neurodegeneration. This hypothesis is supported by recent studies linking neuroinflammatory pathways to PD progression, notably through the mammalian homolog to *tir*-1, SARM1 [30].

Additionally, SARM1, a key player in the axon degeneration pathway in mammals, has been implicated in Parkinson’s disease (PD) through various mechanisms. It operates within a critical balance of axonal NAD^+^ metabolism, where its pro-degenerative actions contribute to the axonal integrity challenges seen in PD [31]. These insights collectively reveal SARM1 as a multifaceted factor in PD and its homologous role in pathogenesis to TIR-1 in *C. elegans*.

It should be noted that miR-71 has only been shown to be highly conserved in invertebrates [28]. There is no evidence that miR-71 or any related sequence is expressed in mammals or humans. However, there is evidence of *SARM1* mRNA being negatively regulated by micro-RNAs in humans [32,33,34]. Additionally, current research has shown that SARM1 is a potential therapeutic target for treating PD and other neurodegenerative disorders [21,35]. Current research has supported that inhibition of SARM1 is neuroprotective in models of nerve disease and injury [36,37]. Our research confirms that the negative regulation of *tir*-1, a homolog of human SARM1, is neuroprotective, adding evidence to the therapeutic potential of SARM1 inhibitors in treating human neurodegenerative disorders.

In conclusion, our study highlights the potential of miR-71 as a key player in neuroprotection against LRRK2-induced neurodegeneration in a *C. elegans* model of Parkinson’s disease. The role of miR-71 in modulating stress responses through *tir*-1/SARM1, particularly in the context of LRRK2 mutations, offers a new avenue for exploring therapeutic interventions. However, while our findings are promising, they are limited to a *C. elegans* model. These findings pave the way for further research into miRNA-based therapies, offering hope for new treatment strategies in PD.

## 4. Materials and Methods

### 4.1. Worm Culturing, Strains, and General Maintenance

All worm strains were maintained and grown at 20 °C on nematode growth medium (NGM) plates seeded with the bacterial *Escherichia coli* strain OP50 as a food source according to established general protocols, unless otherwise stated [38]. Mutant strains were obtained from the specific labs that created them, from the CGC (*C. elegans* Genetics Consortium), or generated in house [39]. All strains that were used in this study are listed in Supplementary Table S1. Double and triple mutant strains were generated using established protocols [40]. To model LRRK2-induced neurodegeneration, we used strains that express LRRK2(G2019S), the most prevalent PD-associated LRRK2 mutation. As a negative control in our neurodegeneration assays, we used a strain that expresses just GFP in dopaminergic neurons (“GFP Control”), to establish a baseline of the normal, wild-type rate of neurodegeneration during aging, as done in previous studies [9,10,41,42]. Note that we could not use a strain expressing wild-type human LRRK2 as a negative control as this expression has been previously shown to also induce neurodegeneration in *C. elegans*, though with a less severe phenotype than strains expressing LRRK2(G2019S) [10,43,44]. Oligonucleotides used in this study for genotyping of mutant strains are listed in Supplementary Table S2.

### 4.2. Basal Slowing Assay

A basal slowing assay measures the difference in motility of worms in the presence of food versus the absence of food. The ability for worms to sense bacterial food is a dopamine-specific behavior which causes the worms to slow down in the presence of food. Each experiment was conducted with a minimum of 20 worms for each strain at each time-point. L1 worms that were age-synchronized were plated on NGM plates seeded with OP50-1 and incubated at 20 °C for 2 days [45]. On days 0, 2, 4, and 6 of adulthood, worms were transferred to a crawl plate, where they were cleaned of food with M9 buffer. They were then transferred to either a plate seeded with food or a clean plate. After 5 min, the worms’ movements were recorded by counting the number of full body bends the worms make in one minute. Percent of slowing response in food was found by calculating the fractional difference in average body bends per minute for each day and strain: ((Average Bends Out of Food) − (Average Bends In Food))/(Average Bends Out of Food) × 100.

### 4.3. Dopaminergic Neurodegeneration Assay

The dopaminergic neurodegeneration assay measures the percentage of healthy dopaminergic neurons in each worm throughout adulthood. L1 worms that were age-synchronized were plated on NGM plates seeded with OP50-1 and incubated at 20 °C for 2 days [25]. On days 0, 2, 5, 7, and 9 of adulthood, a minimum of ten worms from each strain were prepared on a slide using 2% agarose and 5 mM Levamisole. Worms expressing GFP in their dopaminergic neurons (GFP control strains or the LRRK2(G2019S) mutant strains) were imaged by laser scanning confocal microscopy (excitation at 488 nm and detection at 580 nm) at 63× magnification. *C. elegans* contain six dopaminergic neurons in the head region [four cephalic (CEP) neurons and two anterior dierdic (ADE) neurons]. CEP neurons, which are larger and free of interference from endogenous autofluorescence, were scored for presence of an intact cell body and neurites at defined time points throughout adulthood until death [10]. Of note, the co-injection marker, *sur-2*::GFP, in miR-71 overexpressor mutants produces autofluorescence that was distinguished from neuronal fluorescence by identifying neuronal cell bodies attached to axons on microscopy. Data were quantified by comparing the total percentage of healthy neurons (intact cell body and neurites) per strain to the age of the worms (in days of adulthood). The images captured were then merged into z-stack using the ImageJ software (version 2.14.0/1.154f).

### 4.4. qRT-PCR

Isolation of total RNA was performed on day 0 and day 7 of adulthood from all LRRK2 worms and mutants. Experiments for qRT-PCR were done with three biological replicates with about 50 worms per strain per experiment. cDNA synthesis was performed using SuperScript III (Invitrogen, Carlsbad, CA, USA) primed using oligo-dT. Experiments for qPCR (quantitative PCR) were completed using SYBR green and manufacturer protocols for iTaq (BioRad, Hercules, CA, USA). Additionally, miRNA qRT-PCR was performed to detect cel-miR-71 using manufacturer protocols (Assay 001364, ThermoScientific, Waltham, MA, USA). All primers used for qRT-PCR are listed in Supplementary Table S3.

### 4.5. Survival Assay

Worm populations were synchronized via hypochlorite treatment and young adult worms were transferred to NGM plates containing 25 μM fluorodeoxyuridine (FUdR). All experiments were done with three biological replicates with about 40 worms per strain per experiment. Animals were transferred to fresh plates weekly, and viability was scored every 2 days by gentle prodding with a platinum pick. Animals that failed to respond were scored as dead.

### 4.6. Statistical Analysis

Data were presented as the means ± SEM. Statistical significance of differences between results was evaluated using one-way ANOVA followed by the Tukey-Kramer post hoc test for multiple comparisons.

## Data Availability

Data is available upon request to the authors.

## Supplementary Materials

The following supporting information can be downloaded at: https://www.mdpi.com/article/10.3390/ijms25168795/s1, Figure S1: Motility of LRRK2 worms in the absence of food; Figure S2: Expression levels of miR-71 in mutant worms; Figure S3: Expression levels of miR-71 in *tir-1* 3’ UTR mutant worms; Table S1: Strains used in experiments; Table S2: Primer sequences used for genotyping; Table S3: Primer sequences used for qRT-PCR.
