# Supplementary material for: Regulation of TIR-1/SARM-1 by miR-71 Protects Dopaminergic Neurons in a C. elegans Model of LRRK2-Induced Parkinson’s Disease
Source: Int J Mol Sci. 2024 Aug 13;25(16):8795. doi: 10.3390/ijms25168795 (PMC11354575; doi:10.3390/ijms25168795)
Supplement: Supplementary file 1 [file ijms-25-08795-s001.zip › ijms-3068157-supplementary.pdf]

*Supplementary Materials*

# Regulation of TIR-1/SARM-1 by miR-71 Protects Dopaminergic Neurons in a *C. elegans* Model of LRRK2-induced Parkinson's Disease

**Devin Naidoo[1*], Alexandre de Lencastre[2]**

* **Correspondence:** Devin Naidoo: danaidoo@quinnipiac.edu and Alexandre de Lencastre: adelencastre@quinnipiac.edu

## 1 Supplementary Figures and Tables

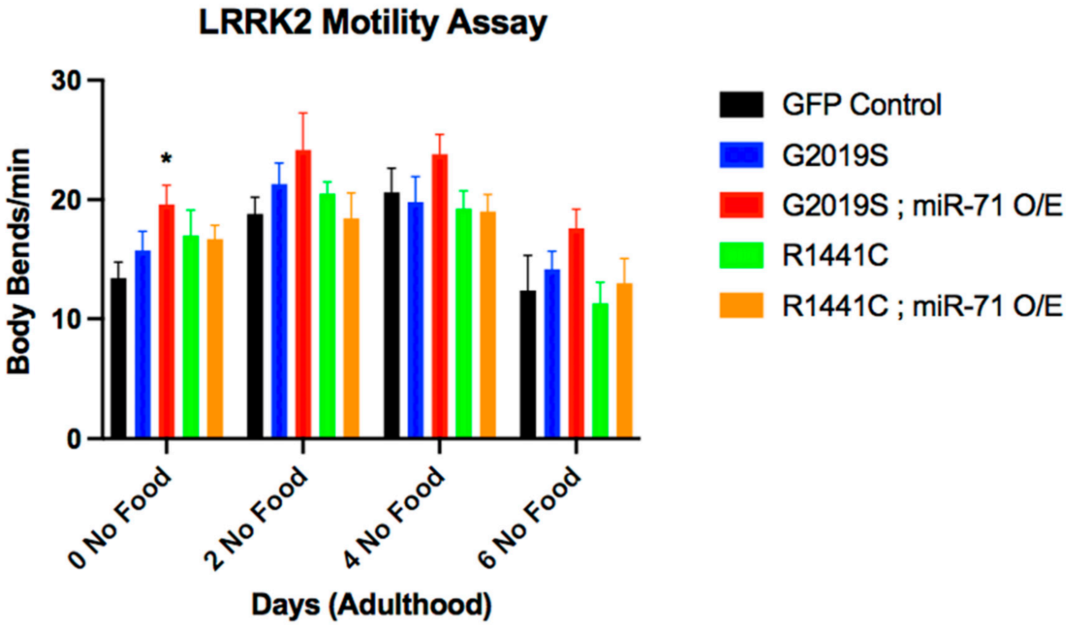


**Supplementary Figure S1: Motility of LRRK2 worms in the absence of food.**

Results from the motility assay of 5 strains, at different time points of adulthood. GFP control represents control worms for LRRK2 mutants, whereas G2019S and R1441C are worms that express mutant forms of human LRRK2 protein in the dopaminergic neurons, in addition to a GFP reporter. On day 0 of adulthood, G2019S ; miR-71O/E worms exhibit increased motility compared to GFP control worms. However, throughout the rest of adulthood, all strains exhibit similar motility to GFP control worms. This suggests that the

loss of basal slowing exhibited by G2019S worms is not associated with any differences in baseline motility of the LRRK2 mutant strains. About 20 animals per strain were used for bending assays. Error bars indicate SEM. One-way ANOVA: *p<0.01 versus GFP control at each time point.

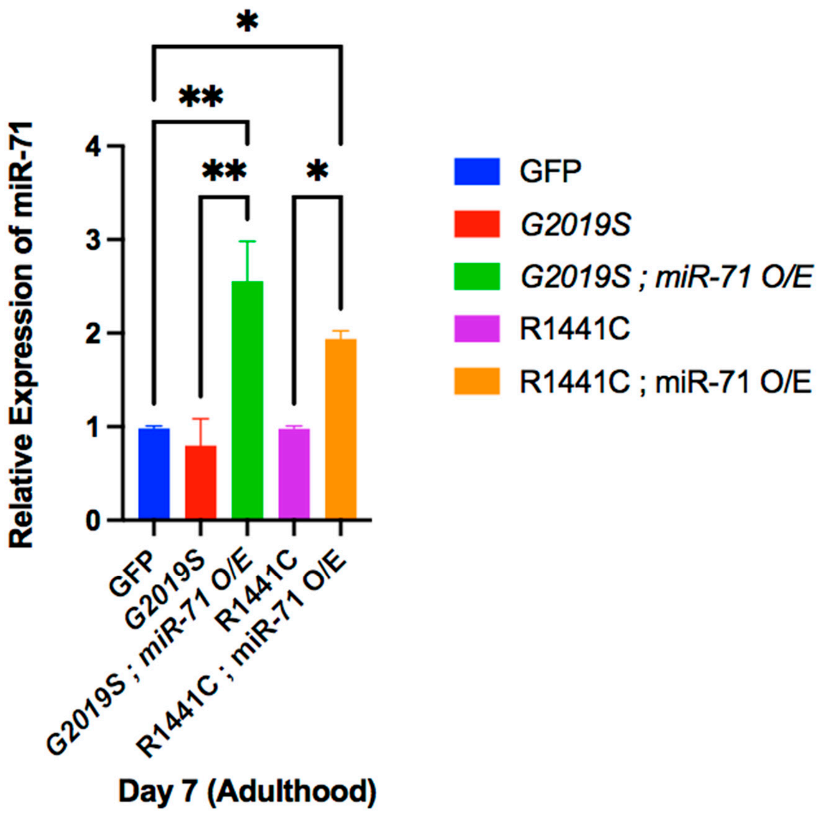


**Supplementary Figure S2: Expression levels of miR-71 in mutant worms.**

Results from qRT-PCR of 5 strains looking at relative expression of miR-71, at day 7 of adulthood. G2019S represents worms that express mutant forms of human LRRK2 protein in the dopaminergic neurons, in addition to a GFP reporter. The experiments for qRT-PCR were done with about 50 worms per strain per experiment ($n = 3$ biological replicates). Error bars indicate SEM. One-way ANOVA: *$p < 0.05$ and **$p < 0.01$.

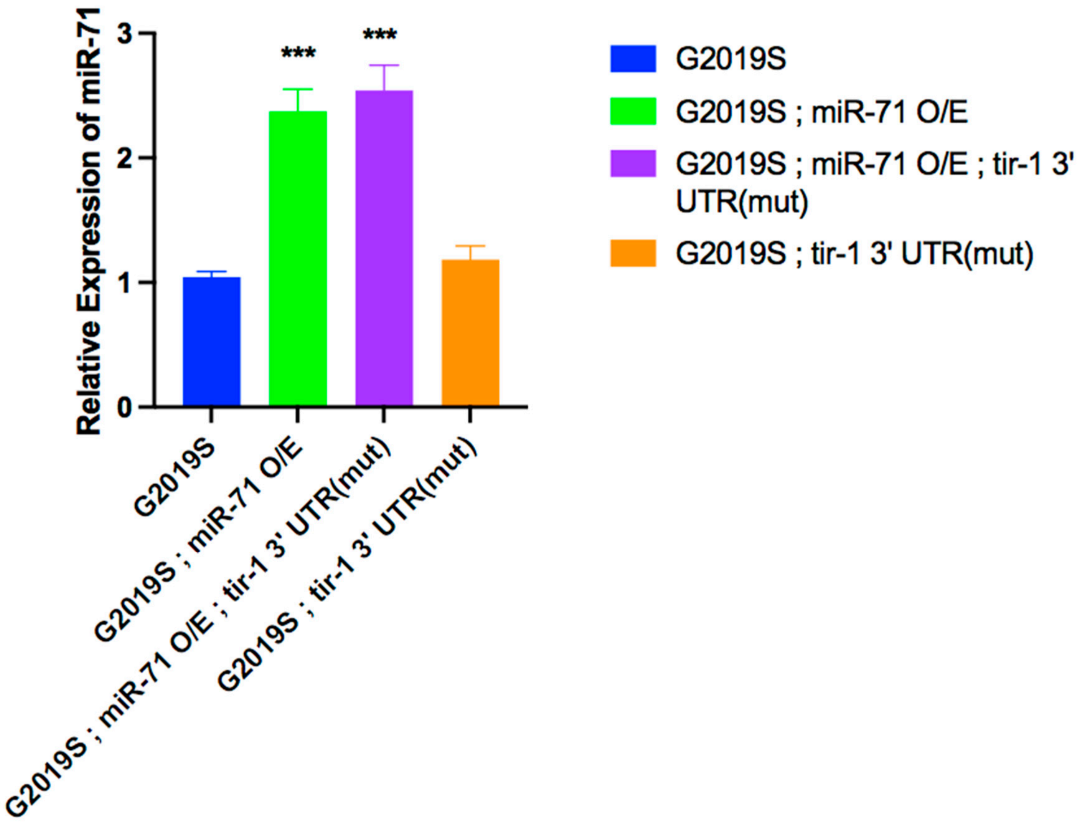


**Supplementary Figure S3: Expression levels of miR-71 in *tir-1* 3′ UTR mutant worms.**

Results from qRT-PCR of 4 strains looking at relative expression of miR-71, at day 7 of adulthood. G2019S represents worms that express mutant forms of human LRRK2 protein in the dopaminergic neurons, in addition to a GFP reporter. Strains crossed with *tir-1* 3′ UTR(mut) represent a loss of three miR-71 binding sites in the *tir-1* 3′UTR. The experiments for qRT-PCR were done with about 50 worms per strain per experiment (n=3 biological replicates). Error bars indicate SEM. One-way ANOVA: ***p<0.005 compared to G2019S.

**Supplementary Table S1: Strains used in experiments.**

| Strain Name | Genotype | Description | Source |
|---|---|---|---|
| N2 | Wild type strain | The wild-type laboratory strain isolated from mushroom compost. | In house. |
| SGC856 | lin-15(n765ts) X ; cwrIs856 [Pdat-1::GFP, Pdat-1::LRRK2(G2019S), lin-15(+)] | Dopaminergic neuron-specific promoter pDat-1 drives expression of human LRRK2 mutant (G2019S) with pDat-1::GFP reporter. | (10) |
| SGC856 ; miR-71 O/E | lin-15(n765ts) X ; cwrIs856 [Pdat-1::GFP, Pdat-1::LRRK2(G2019S), lin-15(+)] ; miR-71 O/E (nls286) ; sur-5::GFP | Cross between SGC856 and miR-71 overexpresser (nls286) with intestinal-specific promoter sur-5::GFP co-injection reporter. | Generated in house. |
| SGC856 ; miR-71 KO | lin-15(n765ts) X ; cwrIs856 [Pdat-1::GFP, Pdat-1::LRRK2(G2019S), lin-15(+)] ; miR-71 KO (n4115) | Cross between SGC856 and miR-71 knockout (n4115). | Generated in house. |

| | | | |
|---|---|---|---|
| SGC730 | lin-15(n765ts) X ; cwrIs730 [Pdat-1::GFP, lin-15(+)] | Dopaminergic neuron-specific promoter pDat-1 drives expression of GFP. “GFP Control”. Control strain for SGC 856. | (10) |
| SGC730 ; miR-71 O/E | lin-15(n765ts) X ; cwrIs856 [Pdat-1::GFP, lin-15(+)] ; miR-71 O/E (nls286) ; sur-5::GFP | Cross between SGC730 and miR-71 overexpresser (nls286) with intestinal-specific promoter sur-5::GFP co-injection reporter. | Generated in house. |
| SGC730 ; miR-71 KO | lin-15(n765ts) X ; cwrIs856 [Pdat-1::GFP, lin-15(+)] ; miR-71 KO (n4115) | Cross between SGC730 and miR-71 knockout (n4115). | Generated in house. |
| SGC856 ; tir-1 KO | lin-15(n765ts) X ; cwrIs856 [Pdat-1::GFP, Pdat-1::LRRK2(G2019S), lin-15(+)] ; tir-1 KO (qd4) | Cross between SGC856 and tir-1 knockout (qd4). | Generated in house. |
| SGC856 ; miR-71 KO ; tir-1 KO | lin-15(n765ts) X ; cwrIs856 [Pdat-1::GFP, Pdat-1::LRRK2(G2019S), lin-15(+)] ; miR-71 KO (n4115) ; tir-1 KO (qd4) | Cross between SGC856 ; miR-71 KO (n4115) and tir-1 knockout (qd4). | Generated in house. |

| | | | |
|---|---|---|---|
| SGC856 ; miR-71 O/E ; *tir-1* 3′ UTR(mut) | lin-15(n765ts) X ; cwrIs856 [Pdat-1::GFP, Pdat-1::LRRK2(G2019S), lin-15(+)] ; miR-71 O/E (nls286) ; sur-5::GFP ; *tir-1* 3′UTR(mut) | Cross between SGC856 and miR-71 overexpressor (nls286) with intestinal-specific promoter sur-5::GFP co-injection reporter. Three binding sites for miR-71 on the *tir-1* 3′UTR have been deleted. | Generated in house with tir-1 3′UTR(mut) received from (15). |
| SGC856 ; *tir-1* 3′ UTR(mut) | lin-15(n765ts) X ; cwrIs856 [Pdat-1::GFP, Pdat-1::LRRK2(G2019S), lin-15(+)] ; *tir-1* 3′ UTR(mut) | Dopaminergic neuron-specific promoter pDat-1 drives expression of human LRRK2 mutant (G2019S) with pDat-1::GFP reporter. Three binding sites for miR-71 on the *tir-1* 3′UTR have been deleted. | Generated in house with tir-1 3′UTR(mut) received from (15). |

**Supplementary Table S2: Primer sequences used for genotyping.**

| Name of Primer | Sequence |
|---|---|
| LRRK2_F1 | CAGGCTGTTAAGACAAGAGC |
| LRRK2_R1 | GGGCACAACCATATTCTTTA |
| MIR-71_nF1 | GTGTTGAGCACTGGATGACG |
| MIR-71_nR1 | GAGCCGATGACTGGAAGGAA |
| MIR-71_nR2 | TCCGACGTCTCACTACCCAT |
| TIR-1_F2 | GGCAGGGCATTGGGTAAATG |
| TIR-1_R2 | ATGCCAGCTGTCAATACCGT |
| TIR-1_R3 | CGCCAACGTGTAAGTTGTTGA |

**Supplementary Table S3: Primer sequences used for qRT-PCR.**

| **Name of Primer** | **Sequence** |
|---|---|
| ACT-1_F1 | CCCCATCAACCATGAAGATC |
| ACT-1_R1 | GACTCGTCGTATTCTTGCTTG |
| TIR-1_F1 | ATGATGATAACTGTGAGGATTGGG |
| TIR-1_R1 | ATAGAAGGCATTTCTTTGGTGG |
| cel-miR-71 | UGAAAGACAUGGGUAGUGA |
